# Temporal Control of Axonal Transport: The Extreme Case of Organismal Ageing

**DOI:** 10.3389/fncel.2019.00393

**Published:** 2019-08-23

**Authors:** Francesca Mattedi, Alessio Vagnoni

**Affiliations:** Department of Basic and Clinical Neuroscience, Maurice Wohl Clinical Neuroscience Institute, IoPPN, King’s College London, London, United Kingdom

**Keywords:** axonal transport, ageing, neurons, mitochondria, *in vivo* imaging, intracellular trafficking, model organisms

## Abstract

A fundamental question in cell biology is how cellular components are delivered to their destination with *spatial* and *temporal* precision within the crowded cytoplasmic environment. The long processes of neurons represent a significant *spatial* challenge and make these cells particularly dependent on mechanisms for long-range cytoskeletal transport of proteins, RNA and organelles. Although many studies have substantiated a role for defective transport of axonal cargoes in the pathogenesis of neurodevelopmental and neurodegenerative diseases, remarkably little is known about how transport is regulated throughout ageing. The scale of the challenge posed by ageing is considerable because, in this case, the *temporal* regulation of transport is ultimately dictated by the length of organismal lifespan, which can extend to days, years or decades. Recent methodological advances to study live axonal transport during ageing *in situ* have provided new tools to scratch beneath the surface of this complex problem and revealed that age-dependent decline in the transport of mitochondria is a common feature across different neuronal populations of several model organisms. In certain instances, the molecular pathways that affect transport in ageing animals have begun to emerge. However, the functional implications of these observations are still not fully understood. Whether transport decline is a significant determinant of neuronal ageing or a mere consequence of decreased cellular fitness remains an open question. In this review, we discuss the latest developments in axonal trafficking in the ageing nervous system, along with the early studies that inaugurated this new area of research. We explore the possibility that the interplay between mitochondrial function and motility represents a crucial driver of ageing in neurons and put forward the hypothesis that declining axonal transport may be legitimately considered a hallmark of neuronal ageing.

## Introduction

**FIGURE 1:**
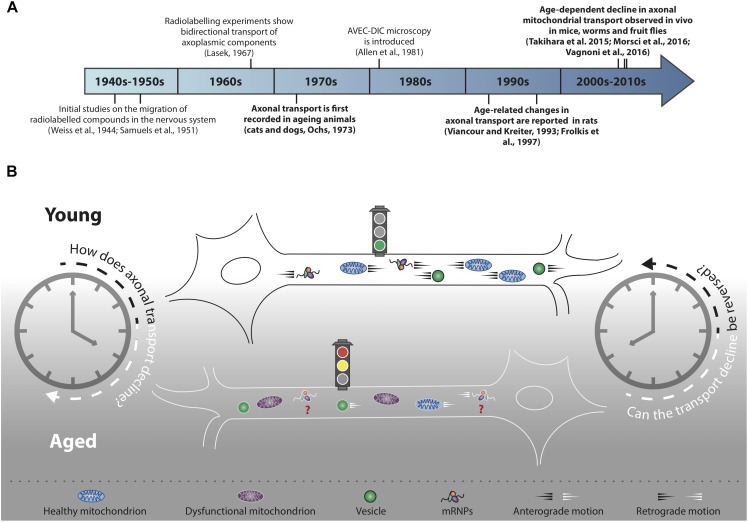
Decline of axonal transport is a hallmark of neuronal ageing. **(A)** Timeline of selected milestones in the study of axonal transport during ageing. In bold, studies of particular interest for the scope of this review. **(B)** Decline of axonal cargo transport during ageing is a common feature across different neuronal populations of several model organisms, although the precise mechanisms of transport decline are not well understood. Not all cargo transport equally declines during ageing (refer to main text for further details) and the motility of many essential cargoes, for instance mRNPs, has not yet been investigated (red question marks). In addition to transport decline, cargoes such as mitochondria undergo an age-dependent functional decay (purple mitochondria). Transport decline correlates with decreased cellular function: can the decline in cargo motility be reversed in an attempt to improve neuronal health during ageing?

Ageing is a process characterised by progressive decline of cellular and organismal functions. How neurons age is a fascinating biological question with vast societal implications, not least due to the ever-increasing life expectancy, but also because ageing is the major risk factor for dementia and other disorders of the nervous system (Niccoli and Partridge, 2012). Neuronal cells heavily rely on mechanisms for the delivery of essential intracellular cargoes within axons and dend- rites; for example, mobilisation of mitochondria to regions of high energy demand, trafficking of mRNA and ribosomal subunits for local translation, signalling endosome-mediated delivery of survival factors and autophagosome-mediated clearance of damaged organelles. As the cellular equivalent of a conveyor belt, when the transport process is interrupted, the functional implications for the neurons are profound. It is therefore not surprising that mutations in genes encoding axonal transport machinery are linked to human disease and that axonal transport impairments have been implicated in the pathogenesis of many neurological diseases (De Vos et al., 2008; Millecamps and Julien, 2013). However, whether this process plays a role in the ageing of neurons has always remained elusive, mainly due to a lack of suitable models to study transport during organismal ageing. Initial studies using radiolabelled amino acids to track axonal transport in neurons of ageing cats and dogs effectively inaugurated a new area of research (Ochs, 1973; Figure 1A). A number of technical advances have recently provided new opportunities to study neuronal trafficking during ageing in different model organisms (Sleigh et al., 2017), thus injecting new life into a largely unexplored research field. In this review, we provide a summary of the current knowledge of intracellular trafficking in ageing neurons, highlighting pressing research questions and new opportunities presented by the latest technical developments.

## Intracellular Trafficking During Neuronal Ageing – A Brief Historical Perspective

Life implies movement and the concept of motion has been essential for our understanding of the physical world since ancient times (Hall, 1965; Kosman, 1969). It was not, however, until the invention of the first single-lensed microscopes that the motion of the microscopic world began to capture the imagination of scientists (Lane, 2015). Further technical advancement, for instance the invention of microscopes with achromatic lenses, started a new era of scientific discoveries that, throughout the 19^th^ century, saw scientists striving to characterise the ‘living jelly’ (gelée vivant, Dujardin, 1835), or ‘living protoplasm’ (Huxley, 1869). The protoplasm, which we now know to be the cytoplasm, was thought to carry all the vital cellular functions and therefore constitute the physical basis of life (Huxley, 1869). Fast forward to the years 1950–1970 to see the first studies carried out in the axons of neurons describing the distribution and migration of cytoplasmic components by classic tracing methods using radiolabelled isotopes (Weiss et al., 1945; Samuels et al., 1951; Lubinska, 1964; Lasek, 1967; Weiss, 1967; Figure 1A). Mammalian nerves, and especially the sciatic nerve, were particularly suitable for this purpose as they are long, contain many axons and can be used to recover abundant radioactive extract to indirectly quantify the progression of the axoplasmic flow. These initial studies paved the way for further work aimed at characterising the transport of cellular components in the nervous system of ageing animals (Ochs, 1973). Ten years later, a monitor was attached to a microscope and AVEC-DIC (video-enhanced contrast, differential interference contrast) microscopy was born (Allen et al., 1981), which provided the first direct, real-time evidence of ‘orthograde’ (i.e., toward the cell periphery) and retrograde (i.e., toward the cell soma) organelle movements in the giant axon of the squid (Allen et al., 1982; Brady et al., 1982).

Over the course of the next two decades, many observations were made suggesting that the movement of specific cargoes within axons may be reduced with age in rodent neurons (Geinisman et al., 1977; Stromska and Ochs, 1982; Brunetti et al., 1987; McQuarrie et al., 1989; Castel et al., 1994; Frolkis et al., 1997; Uchida et al., 2001; Li et al., 2004). These early studies relied mostly on radiolabelling techniques, at times combined with nerve ligation assays (Fernandez and Hodges-Savola, 1994) or the emerging AVEC-DIC microscopy (Viancour and Kreiter, 1993), and effectively opened up a new avenue of research: the study of intracellular trafficking in the nervous system of ageing animals (Figures 1A,B). Because the displacement rates of the axoplasmic flow were generally calculated indirectly by biochemical analyses of the dissected tissue, the spatial and temporal resolution of these experiments was inevitably limited. Distinguishing between cargo-specific and general decline of transport throughout ageing was therefore often challenging. It was only with the discovery of fluorescent proteins, more recently coupled with the development of real-time *in vivo* imaging assays, that a clearer picture has begun to emerge on how neuronal trafficking is orchestrated in ageing organisms.

## Model Organisms: One Model Does Not Fit All

A major bottleneck for ageing studies of neuronal cargo transport has been the lack of suitable models to study this process in real-time during the lifespan of an animal. Substantial progress has recently been made using novel assays in several model organisms, which we briefly review in this paragraph.

### *Mus musculus* (‘The Mouse’)

#### Mean Lifespan of Laboratory Mice: ∼2 Years (Flurkey et al., 2007)

The study of neuronal trafficking in its natural habitat (Lichtman and Fraser, 2001) was significantly boosted by a body of work aimed at clarifying the dynamics of mitochondrial movement in the peripheral nerves of the *MitoMouse*. This transgenic mouse model, in which fluorescent proteins are specifically targeted to the matrix of the neuronal mitochondria, showed that the mitochondria maintain their high dynamicity in adult animals *in vivo* (Misgeld et al., 2007).

More recent studies with the *MitoMouse* have showed that the axonal transport of mitochondria undergoes an age-dependent decline in both retinal ganglion cells (i.e., the CNS) *in situ* (Takihara et al., 2015) and tissue explants of the sciatic nerve (i.e., the PNS) (Gilley et al., 2012; Milde et al., 2015). While the number of motile mitochondria is not overall affected in the mouse CNS up to 25 months of age, their dynamic properties (e.g., duration of movement and distance travelled) progressively decline as the animals age (Takihara et al., 2015). In the PNS *ex vivo*, the fraction of motile mitochondria starts declining already at 6 months of age. This is mirrored by an age-dependent reduction in the transport of the Golgi-derived NMNAT2 vesicles from 3 to 6 months of age in both CNS and PNS explants (Milde et al., 2015). With the exception of reduced vesicular velocity in the PNS tissue explants (Milde et al., 2015), cargo speeds remain largely unaffected by ageing in the mouse nervous system both *in situ* and *ex vivo.* Interestingly, ageing exacerbates the mitochondrial transport defects in tissue explants of a mouse model of tauopathy (Gilley et al., 2012), possibly due to alteration of microtubule dynamics, and in a mouse model of glaucoma *in vivo* (Takihara et al., 2015).

Age-dependent decrease of mitochondrial transport was also observed in dorsal root ganglia neurons (DRGs) cultured from 5-month old mice overexpressing tau P301S, compared to DRGs cultured from younger mice of the same genotype (Mellone et al., 2013). Recent work showed that the biogenesis of the autophagosome decreases in cultured DRGs from aged mice (Stavoe et al., 2019). Combined with evidence that hippocampal neurons cultured from ageing animals display mitochondrial functional decline (Parihar and Brewer, 2007), these findings indicate that *in vitro* cultures of neurons obtained from ageing animals could be used to recapitulate significant aspects of neuronal ageing and complement the *in vivo* work.

In spite of the age-dependent decline in cargo transport observed in different neuronal populations, transport reduction does not seem to be a general feature of neuronal ageing in mice *in vivo*. By performing intravital imaging from the mouse sciatic nerve (Bolea et al., 2014; Gibbs et al., 2016), Sleigh and co-authors showed that trafficking of neurotrophin-containing signalling endosomes is not affected in animals up to 18 months of age (Sleigh and Schiavo, 2016; Sleigh et al., 2018). Signalling endosomes mainly rely on mechanisms for retrograde transport (Schmieg et al., 2014) as opposed to the bidirectional motility of Golgi-derived NMNAT2 vesicles (Milde et al., 2015). The different dynamics of these vesicular cargoes during ageing may therefore reflect inherently distinct dynamic properties. Alternatively, the discrepancy might be due to physiological differences between the *ex vivo* and *in vivo* systems that would impact on specific subset of cargoes. Whatever the case, these findings highlight the need of more work to precisely dissect the dynamics of cargo transport during ageing.

The availability of new techniques to image neuronal cargo transport *in vivo* and *ex vivo* has opened up the possibility to study how intracellular trafficking is regulated in space and time in a more physiological setting relevant for ageing research. These studies so far have mainly focused on the mitochondria and led to refine our understanding of the relationship between mitochondrial dynamics and other aspects of cellular physiology, from neuronal activity (Sajic et al., 2013; Faits et al., 2016) to cellular redox state (Breckwoldt et al., 2014) and disease contexts (Bilsland et al., 2010; Sorbara et al., 2014). Although the mouse experiments have demonstrated the significance of studying transport within an ageing nervous system, this type of study is technically challenging and time consuming. In addition, the cost attached to it is prohibitive for many laboratories and the techniques used remain largely invasive (i.e., dissection could significantly interfere with the physiology of the processes observed). More recently, new work revealed the suitability of the fruit fly *Drosophila melanogaster* and the nematode *Caenorhabditis elegans* to study axonal transport in ageing animals. Due to the shorter lifespan of the invertebrate models, a clear advantage of using these systems is the possibility of making discoveries more rapidly. Key findings obtained in fruit flies and worms could then be validated in mammalian neurons to determine the evolutionary conservation and wider significance of such discoveries.

### *Drosophila melanogaster* (‘The Fruit Fly’)

#### Mean Lifespan of Laboratory Fruit Flies: ∼50 Days (Linford et al., 2013)

The fruit fly is a prime model organism for ageing studies and it has led to seminal discoveries in the ageing field (Clancy et al., 2001; Bjedov et al., 2010). *Drosophila* has been extensively used for studies of axonal transport during larval and pupal developmental stage, providing invaluable insight into the mechanisms regulating neuronal trafficking (Gunawardena and Goldstein, 2001; Guo et al., 2005; Pilling et al., 2006; Wang and Schwarz, 2009; Pathak et al., 2010; Xiong et al., 2010; Avery et al., 2012; Reis et al., 2012; Quraishe et al., 2013; Medioni et al., 2015; Baldwin et al., 2016; Dey et al., 2017). A new assay that exploits the transparent wing of *Drosophila* has recently allowed studying this process in a subset of peripheral neurons of adult animals, with the advantage of monitoring cargo transport as the animals age (Vagnoni and Bullock, 2016). This approach, in which the neurons are imaged from live animals without surgical exposure of the nerve tracts, revealed that the fraction of transported mitochondria, but not their velocity or run length, declines early during ageing (Vagnoni et al., 2016). Halting mitochondrial transport specifically in adult neurons accelerates the appearance of protein aggregation, highlighting the relevance of mitochondrial trafficking for neuronal homeostasis. In this system, the transport of dense core vesicles is largely unaffected during ageing (Vagnoni et al., 2016; Vagnoni and Bullock, 2018). Therefore, as in the mouse sciatic nerve, early decline in axonal transport is not a general feature of ageing, ruling out major disruption to the cytoskeletal network, at least at an early stage. This work also showed that aged neurons are still capable of reversing a prolonged transport decline with an acute intervention in later life by boosting the cAMP/PKA pathway (Vagnoni and Bullock, 2018).

The short lifespan of *Drosophila* makes ageing studies inevitably less challenging than in murine models. Combined with the power of fly genetics, this system warrants fast progress in understanding the mechanisms regulating axonal transport during ageing and the relationship between transport and neuronal function during the animal lifetime. It would be informative to expand these studies to the CNS of flies, for instance by using *ex vivo* explants of adult brains (Ayaz et al., 2008) or the dissected ventral nerve cord (Kudumala et al., 2017), to understand whether decline in mitochondrial transport is shared between different neuronal types.

### *Caenorhabditis elegans* (‘The Worm’)

#### Mean Lifespan of Laboratory Worms: ∼17 Days (Gems and Riddle, 2000)

Since the first DNA mutations correlating with lifespan extension were discovered in *C. elegans* (Klass et al., 1983), a string of discoveries were made in this model organism that challenged the classical view of evolutionary biologists that single mutations could not have a strong effect on lifespan (Kenyon et al., 1993; Morris et al., 1996). As with the fruit fly, the genetic tractability of this organism, coupled with its even shorter lifespan, makes it an excellent model for ageing studies of organelle transport.

Microfluidic devices have been developed for imaging neuronal transport in live worms throughout development and adulthood. By exploiting the translucent nature of these animals, robust transport of Rab3-positive synaptic vesicles can be readily observed in single sensory axons (Mondal et al., 2011). With ageing, the fraction and speed of the vesicles moving in the anterograde direction is decreased in D9 motor neurons and it correlates with impaired synaptic transmission (Li et al., 2016). Mitochondrial transport declines with age in mechanosensory neurons and this is associated with reduced mitochondrial density in the axons and a decreased ability of the animals to respond to oxidative stress throughout ageing (Morsci et al., 2016). Interestingly, by analysing transport in long-lived *daf-2* and *eat-2* mutant worms, the authors show that the prolonged maintenance of mitochondria trafficking is associated with the lifespan extension typical of these animal models and with increased mitochondrial resistance to oxidative stress, thus implicating the insulin-like signalling pathway in the regulation of mitochondrial motility and function during ageing. Redox imbalance has been shown to affect mitochondrial distribution in cultured mammalian cells (Debattisti et al., 2017) and in *Drosophila* adult neurons *in vivo* (Smith et al., 2019). It would be interesting to test whether modulation of cellular stress directly influences mitochondrial transport in this context. Mitochondrial motility is also affected by injury (Rawson et al., 2014) and in a *C. elegans* model of tauopathy (Fatouros et al., 2012), although it is not known whether ageing has any effect on the phenotypes observed.

### *Danio rerio* (‘The Fish’)

#### Mean Lifespan of Laboratory Fish: ∼3.5 Years (Gerhard et al., 2002)

*Danio rerio*, aka zebrafish, has emerged as an important model for studying neuronal function in an organismal context (Walker et al., 2013; Patten et al., 2014), and recent developments have provided powerful assays to measure the transport of mitochondria in a subset of CNS and PNS nerves during development (Plucinska et al., 2012; O’Donnell et al., 2013; Bergamin et al., 2016; Xu et al., 2017; Mandal et al., 2018). These studies have shed light on the distribution of mitochondria within the complex architecture of a neuron *in situ* and have expanded our understanding of the consequences of disease-causing mutations on transport. As with adult fruit flies and worms, the accessibility of zebrafish neurons to microscopy observation is made possible by the translucency of the animal cuticle. This advantage is, however, restricted to developmental stages, limiting ageing studies of organelle transport. Being able to perform live imaging in adult tissues would represent an exciting advancement, for instance by exploiting the see-through nature of the medaka fish (Wakamatsu et al., 2001). The possibility of combining *in situ* imaging of organelle transport with short-lived fish models used in ageing research (Ding et al., 2010; Harel et al., 2015) could have a major impact and provide novel opportunities for the field.

### *Homo sapiens* (‘The Humans’)

#### Mean Life Expectancy of Humans (2015–2020): ∼72 Years (UN DESA, 2017)

The development of new methods in a number of animal models has begun to clarify fundamental mechanisms of cargo transport in ageing neurons and, more broadly, has added to our understanding of the cell biology of the ageing nervous system. It is not straightforward, however, to translate fundamental discoveries attained in model organisms to the ageing process of humans for the benefit of human health. Being able to study this process in a human model of neuronal ageing would help to address this knowledge gap in the field. Human-induced pluripotent stem cells (hiPSC) offer great potential to study intracellular trafficking in neurons derived from patients affected by a range of neurodegenerative diseases. Features associated with cellular age can be induced in these neurons, either by directly interfering with ageing markers or after an extended period in culture of several months (Liu et al., 2011; Ziff and Patani, 2019). Recent developments in stem cell technology have provided novel tools based on cultures of neurons derived directly from fibroblasts of human donors (iNeurons) (Mertens et al., 2015). Unlike hiPSC, these cells are not reprogrammed to an embryonic state. Thus, iNeurons derived from young and old individuals display age-specific transcriptional signatures, along with markers of mitochondrial ageing (Kim et al., 2018), suggesting they are suitable for comparisons of young and old cellular states. A notable advantage of the *in vitro* cellular system is the possibility of carrying out in-depth mechanistic studies that would be more challenging *in vivo.*

Although the fundamental biological insights obtained in model systems *in vitro* and *in vivo* are crucial to advance the field, it is desirable to validate these findings in what arguably remains the best *in vivo* model, the humans themselves. Imaging approaches using manganese-enhanced magnetic resonance imaging (Mn^2+^-MRI) have revealed a decrease of the bulk transport in the olfactory system of aged mice (Cross et al., 2008). This non-invasive approach could potentially be translated to humans, with the possibility of recording transport rates in longitudinal studies from living human brains. These advantages, however, need to be weighed against the potential toxicity of Mn^2+^ and the inevitable lack of tracer specificity which only reports on the bulk axoplasmic flow. Alternatively, pulse doses of non-radioactive tracers, such as heavy water (^2^H_2_O), could be followed by cerebrospinal fluid (CSF) sampling in young and old individuals through lumbar puncture and mass spectrometric analysis of the labelled proteins (Fanara et al., 2012). CSF-based biomarkers of neuronal cargo transport could then be identified to monitor defective transport during ageing. More recently, an exciting approach combining adaptive optics and optical coherence tomography (AO-OCT) has been applied to imaging fine neuronal details in the human retina *in vivo* (Liu et al., 2017). Enhanced visualisation of single axonal fibres (Gray et al., 2008), combined with the topical application of non-toxic chemical markers of subcellular structures, would open up the unprecedented opportunity to peer into the intracellular dynamics of the ageing nervous system of humans.

## Mitochondria, an Atypical (But Essential) Cargo

Mitochondria have a central role in governing energy metabolism via ATP production and are regarded as the ‘powerhouse of the cell’ (Siekevitz, 1957). Their fundamental role extends to numerous other processes essential for life, including fatty acid beta-oxidation, calcium signalling and steroid synthesis, and mitochondrial dysfunction is widely regarded as one of the hallmarks of ageing (Bishop et al., 2010; López-Otín et al., 2013; DiLoreto and Murphy, 2015).

Many studies have focused on the mechanisms regulating mitochondrial motility and showed that disruption of mitochondrial transport invariably results in altered neuronal function (Sheng and Cai, 2012). However, mitochondria are atypical axonal transport cargoes as most of them can remain stationary at any given time (Hollenbeck and Saxton, 2005; Gutnick et al., 2019). Despite their lack of motility relative to other cargoes, reduction in mitochondrial movements is linked to decreased cellular health during ageing and disease, and declining transport appears to be an evolutionary conserved hallmark of ageing across several neuronal subtypes, from invertebrates to mammals (Takihara et al., 2015; Morsci et al., 2016; Vagnoni et al., 2016). On the other hand, upregulation of mitochondrial transport, for instance by boosting the cAMP-PKA pathway, correlates with increased cellular health in aged *Drosophila* neurons (Vagnoni and Bullock, 2018) and underlies synapse formation in Aplysia neurons (Badal et al., 2019). In fruit flies and worms, upregulation of mitochondrial transport also correlates with the suppression of Wallerian degeneration (Avery et al., 2012) and is protective against axotomy-triggered degeneration (Rawson et al., 2014). It has also been shown that transport upregulation *in vitro* is necessary for the maintenance of neuronal homeostasis after injury (Zhou et al., 2016) and is sustained after regeneration in young and old mice *ex vivo* (Mar et al., 2014; Milde et al., 2015). It would be interesting to expand these studies in ageing animals *in vivo*, for instance by exploiting current assays for imaging the mouse spinal cord after injury (Kerschensteiner et al., 2005; Davalos et al., 2008; Farrar et al., 2012). Increasing mitochondrial trafficking may not, however, always represent a viable option for improving cellular health (Zhu and Sheng, 2011; Faits et al., 2016) suggesting context-specific functional outcome of transport upregulation.

## How is Mitochondrial Distribution Maintained Throughout Ageing? The Problem of ‘Transport Imbalance’

The advent of systems for *in vivo* imaging of organelle transport have, on one hand, provided new platforms to explore open questions in the field and, at the same time, presented us with new challenging questions (Table 1). On the other hand, these new methodologies have validated the general principles governing neuronal trafficking discovered in cultured neurons and expanded them to an *in vivo* setting. There are striking similarities in the properties of mitochondrial transport between *in vitro* and *in vivo* systems. For instance, more organelles are consistently transported in the anterograde than in the retrograde direction, both in young and old neurons (Vagnoni et al., 2016; Misgeld and Schwarz, 2017). What happens to the mitochondria that, seemingly, do not make the trip back along the axon is an open question and whether interfering with the anterograde/retrograde ratio has an impact on ageing is unknown. It is still unclear if the ‘transport imbalance’ may be coupled to the recycling of the organelles (Miller and Sheetz, 2004; Maday and Holzbaur, 2014; Lin et al., 2017) with *in vitro* studies suggesting that motile mitochondria may become anchored at sites of functional requirement such as synapses or nodes of Ranvier (Chang et al., 2006; Obashi and Okabe, 2013; Lewis et al., 2016; Gutnick et al., 2019). A notable exception are the ALM sensory neurons of *C. elegans* where mitochondria are transported with a retrograde bias throughout life (Morsci et al., 2016). Interestingly, these neurons lack synaptic connections, suggesting that synaptic activity may play a crucial role in determining the anterograde transport bias of mitochondria in mature neurons. Whatever the mechanism accounting for the majority of mitochondria constantly travelling in the anterograde direction, this is an evolutionary conserved feature likely to support a fundamental cellular demand.

**TABLE 1:** Outstanding and new questions facing the field of neuronal trafficking during ageing.

Outstanding	What are the functional consequences of transport decline of specific cargoes?
	Can we tease out cell-autonomous from non-cell-autonomous mechanisms affecting cargo transport?
	Is the cytoskeleton structure rearranged during ageing? If so, how does this impact transport?
	Do specific post-translational modifications of key components of the transport machinery (e.g., motors, adaptors, microtubules, microtubule-associated proteins, and regulatory kinases) affect cargo trafficking during ageing?
	How does a neuron sustain the transport imbalance of certain cargoes (i.e., many more cargoes continously transported with an anterograde bias)?
New	What is the functional relevance of the timing of cargo decline during ageing (i.e., early vs. late decline)?
	Are specific subsets of neurons more sensitive to age-dependent transport decline that could predispose them to degeneration, hence contributing to neuronal specificity in some neurodegenerative conditions?
	Are neurons able to sustain a prolonged upregulation of cargo transport, either through genetic or chemical modification, that could alleviate age-related dysfunction?
	What is the composition of the cytoplasmic milieu in ageing cells and how does this affect cargo transport?
	Does the architecture and recruitment of the cargo transport machinery change during ageing? Does the changing abundance of motor and adaptor proteins favour the formation of specific transport complexes that are less likely to form in early life?

Imbalanced mitochondrial biogenesis (López-Lluch et al., 2008; Seo et al., 2010) and changes in mitochondrial fission and fusion (Cao et al., 2017) were observed during ageing, although a mechanistic association with mitochondrial transport and distribution is still unclear at this stage. It is not known whether mitochondrial anterograde bias throughout life and age-dependent transport decline lead to toxic accumulation in the soma or to a depletion of the organelle over time (Morsci et al., 2016). This is an open question which deserves further investigation.

A progressive and specific decline in the motility of mitochondria has been observed during developmental maturation of cortical axons in live mice up to 45 days of age (Lewis et al., 2016; Smit-rigter et al., 2016) and in maturing dendrites of ganglion cells of retinal explants (Faits et al., 2016). The general reduction in motility is, in these cases, necessary to distribute the organelles by stably anchoring them at specific cellular sites. In this light, it is tempting to speculate that decline in mitochondrial transport in mature neurons may be part of a run-on of developmental processes (Azpurua et al., 2018; Ezcurra et al., 2018). Thus, early transport decline, beneficial during development, may become detrimental in later life when the functional demand of a neuron changes, by contributing to age-dependent decline of neuronal functions such as reduction of ATP production (Kim et al., 2018), impaired calcium homeostasis (Nikoletopoulou and Tavernarakis, 2012) and increased oxidative stress (Castelli et al., 2019). Choosing the appropriate window of time to upregulate the transport therefore appears crucial to be able to meaningfully impact on neuronal health in later life (Vagnoni and Bullock, 2018).

## Outlook

**FIGURE 2:**
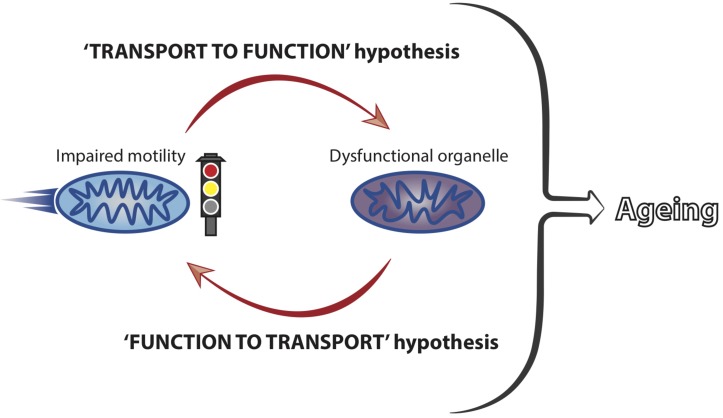
Mitochondrial motility and function are coupled and influence neuronal ageing. Mitochondrial transport and function are closely linked and we hypothesise that impaired motility leads to a loss of organellar functionality. Dysfunctional mitochondria, in turn, can affect their own motility, with a net result of locking the organelles into a vicious cycle of transport-function impairment. This is predicted to culminate in the accumulation of stalled and dysfunctional mitochondria, thus significantly contributing to the ageing process of neurons by failing to meet the functional demands of ageing cells.

In spite of the strong association between axonal transport and the maintenance of neuronal health, it is unclear how modulation of transport mechanistically affects neuronal ageing phenotypes. Mitochondria are the best characterised cargoes in transport studies during ageing and their transport and function are closely linked (Saxton et al., 2012). It is therefore conceivable that an early decrease in motility could trigger a rapid and dysfunctional response from the mitochondria (‘transport to function’ hypothesis, Figure 2). This may represent a causal event at the beginning of a damaging cascade leading to late onset neuronal dysfunction.

Mitochondria accumulate damage over time (Bratic and Larsson, 2013; Payne and Chinnery, 2015; Sun et al., 2016) and it has been suggested that mitochondrial dysfunction leads to transport reduction (Miller and Sheetz, 2004; Wang et al., 2011; Hwang et al., 2014; Grimm et al., 2018). Therefore, it is also possible that early mitochondrial damage could induce transport decline in early life (‘function to transport’ hypothesis, Figure 2). Reduced transport may, in turn, trigger a deleterious functional response in a vicious cycle that would ultimately lead to cellular dysfunction with increased age (Figure 2). Increased abundance of proteins involved in mitochondria function has been correlated to cognitive stability during human ageing and it would be interesting to understand whether these molecules impact on mitochondrial transport in later life (Wingo et al., 2019). Future studies should address in more detail the precise temporal cascade of events linking decreased motility to cellular dysfunction. Combining the knowledge derived from different model systems is predicted to facilitate our understanding of how mitochondrial transport is integrated within other cellular processes to affect ageing of neurons.

Gaining deeper insight into the mechanisms controlling age-dependent failure of cargo transport, including expanding the repertoire or cargoes imaged, might prove critical to understand how neuronal health declines in ageing organisms (Table 1). Early impairments to regulatory nodes of intracellular trafficking could represent the initial hits of a multi-hit model of neurodegeneration (Sulzer, 2007; Garden and La Spada, 2012). In this view, it becomes a priority to identify early phenotypes at the beginning of a cascade of events leading to neuronal dysfunction. Recognising viable targets to restore cargo transport during ageing may therefore prove essential. Motor abundance (Puthanveettil et al., 2008; Li et al., 2016; Vagnoni and Bullock, 2018; Badal et al., 2019) and the activity of signalling pathways linked to lifespan extension (Li et al., 2016; Morsci et al., 2016; Vagnoni and Bullock, 2018) are potential candidates, even though these studies are limited to only few cargoes. More work is needed to understand the general mechanisms governing intracellular trafficking during ageing in a bid to target axonal transport, in conjunction with other cellular processes (Riera and Dillin, 2015), to improve the health of neurons during ageing (Figure 1).

## Author Contributions

AV wrote the initial draft of the manuscript with significant contribution from FM. AV and FM revised and approved the manuscript.

## Conflict of Interest Statement

The authors declare that the research was conducted in the absence of any commercial or financial relationships that could be construed as a potential conflict of interest.
